# Speeding Up the Heart? Traditional and New Perspectives on HCN4 Function

**DOI:** 10.3389/fphys.2021.669029

**Published:** 2021-05-27

**Authors:** Konstantin Hennis, René D. Rötzer, Chiara Piantoni, Martin Biel, Christian Wahl-Schott, Stefanie Fenske

**Affiliations:** ^1^Center for Drug Research, Department of Pharmacy, Ludwig-Maximilians-Universität München, Munich, Germany; ^2^Institute for Neurophysiology, Hannover Medical School, Hanover, Germany; ^3^German Center for Cardiovascular Research (DZHK), Partner Site Munich Heart Alliance, Munich, Germany

**Keywords:** sinoatrial node, pacemaking, chronotropic effect, heart rate regulation, autonomic nervous system, HCN4 channel, cyclic nucleotide-gated (HCN) channels, hyperpolarization-activated cation channel

## Abstract

The sinoatrial node (SAN) is the primary pacemaker of the heart and is responsible for generating the intrinsic heartbeat. Within the SAN, spontaneously active pacemaker cells initiate the electrical activity that causes the contraction of all cardiomyocytes. The firing rate of pacemaker cells depends on the slow diastolic depolarization (SDD) and determines the intrinsic heart rate (HR). To adapt cardiac output to varying physical demands, HR is regulated by the autonomic nervous system (ANS). The sympathetic and parasympathetic branches of the ANS innervate the SAN and regulate the firing rate of pacemaker cells by accelerating or decelerating SDD–a process well-known as the chronotropic effect. Although this process is of fundamental physiological relevance, it is still incompletely understood how it is mediated at the subcellular level. Over the past 20 years, most of the work to resolve the underlying cellular mechanisms has made use of genetically engineered mouse models. In this review, we focus on the findings from these mouse studies regarding the cellular mechanisms involved in the generation and regulation of the heartbeat, with particular focus on the highly debated role of the hyperpolarization-activated cyclic nucleotide-gated cation channel HCN4 in mediating the chronotropic effect. By focusing on experimental data obtained in mice and humans, but not in other species, we outline how findings obtained in mice relate to human physiology and pathophysiology and provide specific information on how dysfunction or loss of HCN4 channels leads to human SAN disease.

## Introduction

### Anatomy and Structure of the Sinoatrial Node and Sinoatrial Node Network

**FIGURE 1:**
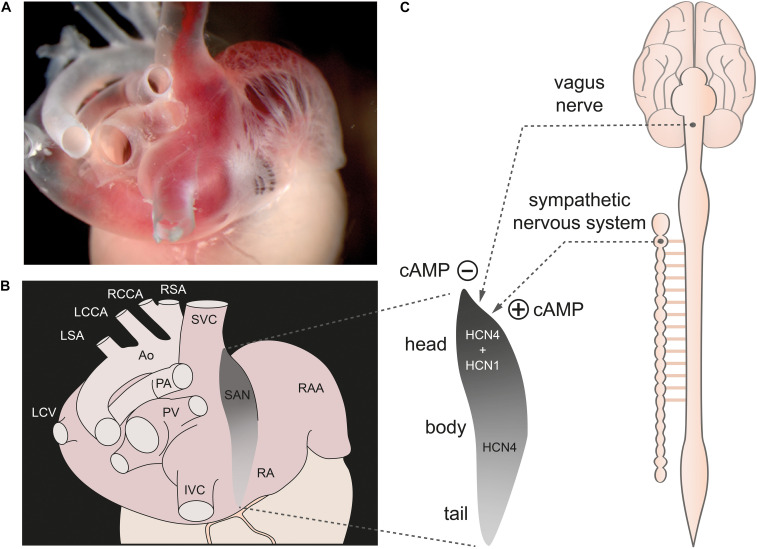
The sinoatrial node. **(A)** Dorsolateral view of the sinoatrial node region of a gelatine-filled mouse heart. **(B)** Schematic illustration of the heart shown in panel **(A)**, depicting the location of the sinoatrial node (SAN) (gray) within the right atrium (RA). **(C, left)** Magnification of the SAN region. The cranial portion is referred to as the sinus node “head,” the middle portion as the “body,” and the caudal portion as the “tail.” HCN1 channels are only expressed in the head region whereas HCN4 channels are expressed throughout the whole SAN. **(C, right)** The SAN is innervated by the sympathetic and parasympathetic nervous system (dashed lines). Activity of both ANS branches tightly controls cAMP concentration in SAN cells. Abbreviations: Ao, Aorta; IVC, inferior vena cava; LCCA, left common carotid artery; LCV, left cranial vein; LSA; left subclavian artery; PA, pulmonary arteries; PV, pulmonary veins; RAA, right atrial appendage; RCCA, right common carotid artery; RSA, right subclavian artery; SVC, superior vena cava. Figure is adapted from Hennis et al., 2021.

The sinoatrial node (SAN) is a spindle-shaped structure located at the posterior side of the right atrium of the heart (Figure 1). It runs from the superior vena cava along the sulcus terminalis toward the inferior vena cava (Liu et al., 2007). The cranial portion is referred to as the sinus node “head,” the middle portion as the “body,” and the caudal portion as the “tail” (Sanchez-Quintana et al., 2005). In the mouse, the SAN is extremely small with a longitudinal dimension along the crista terminalis of about 500–1000 μm and a width of approximately 150 μm (Verheijck et al., 2001; Liu et al., 2007). In humans, the dimensions of the SAN are naturally larger with a length along the crista terminalis of approximately 14–15 mm, a width of 6–7 mm, and a thickness of about 1 mm (Fedorov et al., 2010).

**FIGURE 2:**
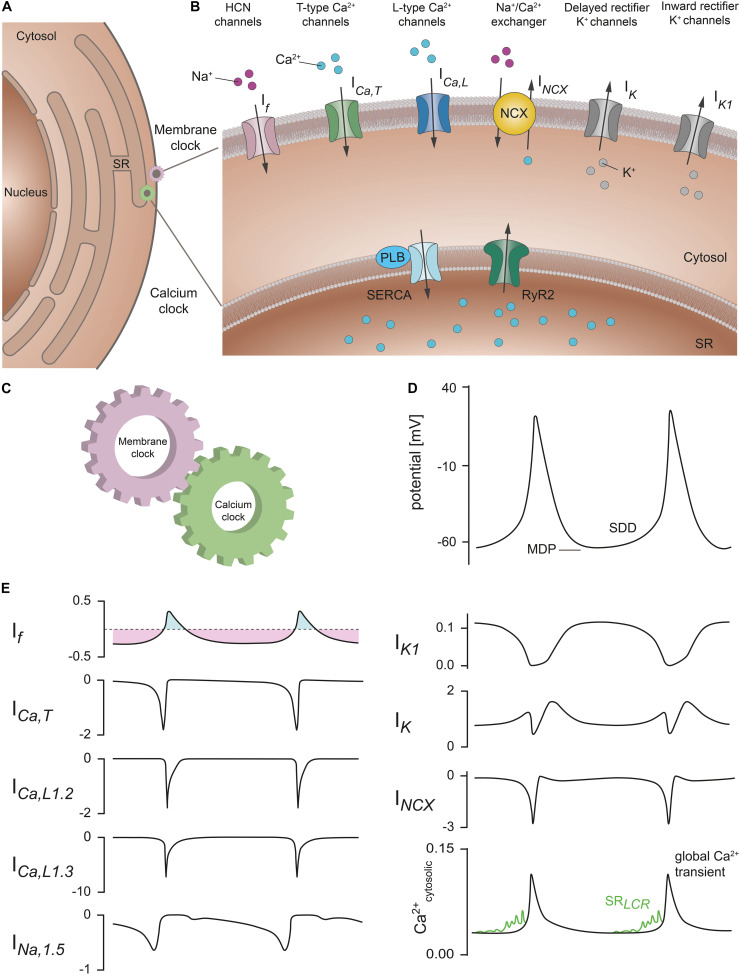
Membrane and calcium clock contribute to the generation of SDD. **(A)** Schematic representation of a SAN cell containing the nucleus, cytosol, and sarcoplasmic reticulum (SR). **(B)** Section of the cell with a magnified view of the plasma membrane and SR membrane. Various proteins that contribute to SDD and are localized in the plasma membrane are collectively described as the *membrane clock*. These include hyperpolarization-activated cyclic-nucleotide gated (HCN) cation channels (pink), T-type Ca^2+^ channels (green), L-type Ca^2+^ channels (blue), Na^+^/Ca^2+^ exchanger proteins (NCX, yellow), rapid and slow delayed rectifier K^+^ channels (gray), and inward rectifier K^+^ channels (gray). The corresponding ionic currents (I) are indicated. Intracellular Ca^2+^ cycling events that contribute to the pacemaker process are summarized as *calcium clock*. Among them are sarco-/endoplasmic reticulum Ca^2+^ ATPases (SERCA) (light blue) associated with the regulatory protein phospholamban (PLB) and ryanodine receptors (RyR2) (dark green) located in the SR membrane. **(C)** Functional interaction of the membrane clock and calcium clock is required to ensure regular and rhythmic excitation of the cells. **(D)** Spontaneous SAN action potentials with the characteristic slow diastolic depolarization phase (SDD). The maximum diastolic potential (MDP) is indicated. **(E)** Relative contribution of the ionic currents responsible for SDD and spontaneous action potential firing in mouse SAN cells according to the mathematical model published by Kharche et al. (2011). Current amplitudes are normalized to the cell capacitance (in units of pA/pF). (Upper left panel) I_f_ manifests as a mainly time-independent, but bidirectionally flowing current. The inward (light purple) and outward component (light blue) are indicated. (Lower right panel) Sarcoplasmic reticulum local calcium releases (SR_*LCR*_) (green) that characteristically occur during late SDD have been added to the model data for cytosolic Ca^2+^ transients according to Lakatta et al. (2010). Abbreviations: I_f_, hyperpolarization-activated cyclic nucleotide-gated (HCN) current; I_Ca,T_, T-type Ca^2+^ current; I_Ca,L1.2_, L-type Ca^2+^ channel isoform Ca_v_1.2 current; I_Ca,L1.3_, L-type Ca^2+^ channel isoform Ca_v_1.3 current; I_Na,1.5_, Na^+^ channel isoform Na_v_1.5 current; I_K1_, inward rectifying K^+^ current; I_K_, delayed rectifying K^+^ current; I_NCX_, Na^+^/Ca^2+^ exchanger current; Ca^2+^_cytosolic_, cytosolic Ca^2+^ concentration; LCR, local calcium release. For further details see text. Figure is adapted from Lakatta et al. (2010), Kharche et al. (2011), Cingolani et al. (2018).

The SAN network is composed of different cell types comprising spontaneously active pacemaker cells that are interspersed with fibroblasts and embedded within a matrix of fibrous connective tissue, predominately consisting of elastin and collagen (Monfredi et al., 2010; Ho and Sanchez-Quintana, 2016). Pacemaker cells are electrically coupled to each other via gap junctions. In addition, there are electrical connections between pacemaker cells, atrial cardiomyocytes and macrophages residing in the SAN. The pacemaker cells also have synaptic contacts with the nerve endings of the sympathetic nerve and vagus nerve, through which the activity of the cells can be changed and controlled (Verheijck et al., 2002; Camelliti et al., 2004; Pauza et al., 2014; Hulsmans et al., 2017). It is now known that the functional interactions of individual pacemaker cells in this cellular network with each other and with the other cell types in the network are of general importance for electrical synchronization to a common electrical rhythm of the sinus node. The slow diastolic depolarization (SDD) is a unique feature of pacemaker cells that drives generation of spontaneous and rhythmic action potentials (Figure 2). After completion of the repolarization, pacemaker cells in the SAN do not remain at a stable resting membrane potential but instead slowly depolarize the membrane toward a threshold potential at which the next action potential is generated (Figure 2D). The slope of SDD essentially determines the duration of the pacemaker cycle and thus the heart rate (HR).

### HCN Channels Are Markers for the SAN

Marker proteins for pacemaker cells within the SAN are hyperpolarization-activated cyclic nucleotide-gated (HCN) channels, of which four isoforms (HCN1-4) exist in mammals. HCN channels belong to the superfamily of voltage-gated cation channels. Four HCN subunits are assembled around a central ion-conducting pore. Each of the subunits consists of six α-helical transmembrane segments (S1-6) and the intracellular N- and C-termini. HCN channels are opened by hyperpolarization and are the molecular determinants of the ionic current I_f_ in pacemaker cells (Ludwig et al., 1998; Mangoni and Nargeot, 2008; Biel et al., 2009; Li et al., 2015). The channel properties can be modulated by binding of the cyclic nucleotide cyclic adenosine monophosphate (cAMP) to a cyclic nucleotide-binding domain (CNBD) in the C-terminus of the channel (Zagotta et al., 2003), thereby facilitating channel opening. The different isoforms are characterized by some different features, such as kinetics, voltage dependence and cAMP modulation. For example, HCN4 displays the slowest activation and deactivation kinetics and opens at more negative potentials than the other isoforms (Wahl-Schott and Biel, 2009). In contrast, HCN1 displays the fastest kinetics and opens at more positive potentials. Furthermore, HCN4 is the most sensitive to the second messenger cyclic AMP, while the subtype HCN1 is only weakly affected by cAMP (Wahl-Schott and Biel, 2009).

In the mouse, HCN4 is the predominant isoform expressed in pacemaker cells throughout the SAN, whereas HCN1 channels are expressed only in the head region (Fenske et al., 2013). HCN2 channels are expressed only very weakly and are anatomically restricted to the periphery of the SAN (Liu et al., 2007; Herrmann et al., 2011; Fenske et al., 2013).

In humans, HCN4, HCN1, and HCN2 channels are expressed uniformly throughout the SAN without any of the isoforms being restricted to a particular region (Li et al., 2015). Importantly, HCN1 is almost exclusively expressed in the SAN, while HCN2 and HCN4 are present both in SAN pacemaker cells and surrounding right atrial myocytes. Therefore, despite being the predominant isoform also in human SAN, HCN4 cannot be used as a unique marker to identify human SAN pacemaker cells (Chandler et al., 2009; Kalyanasundaram et al., 2019).

Since its first discovery, the I_f_ current and its role in cardiac pacemaking have been highly debated, leading to controversial views on the importance of I_f_ in pacemaker activity. On the one side, several groups have questioned a direct involvement of I_f_ in the generation of action potentials because of its negative threshold of activation and slow time constant (Noma et al., 1980; Yanagihara and Irisawa, 1980), suggesting that the purpose of the current is more likely to maintain a low membrane potential in pacemaker cells. Furthermore, Noma and collaborators showed that Cs^+^ reversibly blocks I_f_ but does not significantly affect the rate of pacemaking (Noma et al., 1983). On the other side, Denyer and Brown (1990) strongly suggested that the I_f_ current normally makes an important contribution to the depolarization of all SAN pacemaker cells, while the groups of Kreitner (1985); Nikmaram et al. (1997) described a possible different role of I_f_ in different SAN regions with a greater influence in the periphery than in the center of the SAN.

## Spontaneous Activity of Sinoatrial Node Pacemaker Cells

The ability of SAN pacemaker cells to generate SDD and spontaneous action potentials has been attributed to the interplay of two major cellular mechanisms named *membrane clock* and *calcium clock* (Figures 2A–C; Lakatta et al., 2010). Insights into these mechanisms are derived from experimental data obtained in mice, which are also the basis for mathematical models of action potential firing in mouse SAN cells (Figures 2D,E; Kharche et al., 2011).

The *membrane clock* comprises the activity of all ion channels and transporters that are localized in the cell membrane and contribute to the membrane potential characteristics of SDD (Figures 2D,E). Following termination of an action potential, when the membrane potential is most negative (maximum diastolic potential), the early phase of SDD is initiated by the depolarizing inward current I_f_ that is mediated by constitutively open HCN channels and persists throughout the range of SDD (DiFrancesco et al., 1986; DiFrancesco, 1993; Mangoni and Nargeot, 2008; Biel et al., 2009). The I_f_ current drives the membrane potential toward the threshold potential at which voltage-gated T-type (Ca_V_3.1) and L-type (Ca_V_1.3) Ca^2+^ channels are activated. In addition, voltage-gated sodium channels (I_Na,1.5_, Figure 2E) contribute to membrane depolarization during late SDD and the action potential upstroke. Sodium currents have been shown to be involved in both pacemaking and impulse conduction within the SAN of mice as well as humans, although there seem to be profound species-dependent differences regarding the contribution of different sodium channel isoforms (Lei et al., 2004, 2007; Li et al., 2020). The combination of all inward currents further depolarizes the membrane during late SDD, which leads to an additional opening of voltage-gated L-type Ca_V_1.2 channels. I_Ca,*L*_ mainly generates the action potential upstroke and is responsible for coupling excitation to contraction (electromechanical coupling): Ca^2+^ entering cardiomyocytes via Ca_V_1.2 activates ryanodine receptor 2 (RyR2) which initiates global intracellular Ca^2+^ release from the sarcoplasmic reticulum (SR) (calcium-induced calcium release) and triggers myofibril contraction. Depolarization of the membrane inactivates voltage-gated calcium currents and activates delayed rectifier potassium channels, which conduct the outward currents I_K,r_ and I_K,s_ that are responsible for membrane repolarization and action potential termination (Figure 2B,E; Mangoni and Nargeot, 2008; Mesirca et al., 2021). At the beginning of the following pacemaker cycle, the decay of outward potassium currents due to time- and voltage-dependent inactivation of I_K,r_ and I_K,s_ allows the inward pacemaker currents to depolarize the membrane and thus represents another key component of early SDD (Irisawa et al., 1993). Moreover, the inward rectifier potassium current I_K1_ is prominently expressed in murine SAN cells (Figures 2B,E), whereas I_K1_ is small or absent in human SAN (Chandler et al., 2009). This might be a reason why membrane potentials in human SAN cells are more positive, which would indirectly support pacemaking (Dobrev, 2009).

In addition to the *membrane clock*, intracellular Ca^2+^ cycling events take place that may significantly contribute to the pacemaker process and are summarized as *calcium clock* (Figures 2A–C; Lakatta et al., 2010). During late SDD, periodically occurring, rhythmic local calcium release events (LCRs) from the SR are mediated by spontaneous opening of RyR2 (Vinogradova et al., 2004). The resulting increase in the intracellular Ca^2+^ concentration activates the sodium-calcium exchanger (NCX), a transporter located in the cell membrane that extrudes one Ca^2+^ ion out of the cell in exchange for three Na^+^ ions entering the cell when operating in its forward mode (Figures 2B,E; Mangoni and Nargeot, 2008). Since in cardiac myocytes the reversal potential of NCX is about −20 mV (Bers et al., 1988; Baartscheer et al., 2011), the outward transport of Ca^2+^ coupled to inward transport of Na^+^ (forward mode) is favored in the diastolic range of membrane potentials. This causes a net inward current (I_NCX_) which further depolarizes the membrane and is responsible for the exponential increase in membrane potential during the late phase of SDD, immediately prior to the action potential upstroke (Figures 2D,E; Bogdanov et al., 2006). The extent of LCRs critically depends on SR Ca^2+^ load, which in turn is regulated by the activity of the sarco-/endoplasmic reticulum Ca^2+^ ATPase (SERCA) that refills the SR Ca^2+^ stores after action potential termination (Vinogradova et al., 2010). The Ca^2+^ reuptake into the SR is significantly regulated by phospholamban, a 52-amino acid peptide directly inhibiting SERCA activity (Vinogradova et al., 2018). This *calcium clock* concept was mainly derived from confocal calcium imaging experiments in isolated cells of the SAN. Recently, it was strengthened by combined calcium imaging and electrophysiological experiments in human primary SAN pacemaker cells (Tsutsui et al., 2018). Together, the proper function of both the *membrane clock* and *calcium clock* processes as well as functional coupling of the underlying mechanisms are indispensable for the pacemaker process (Tsutsui et al., 2018). In addition, several proteins associated with the *membrane clock* or *calcium clock* are modulated by activity of the autonomic nervous system (ANS). Therefore, there are many potential candidates that could be involved in or mediate the chronotropic effect and HR regulation.

## Does cAMP-Dependent Regulation of HCN4 Mediate the Chronotropic Effect?

**FIGURE 3:**
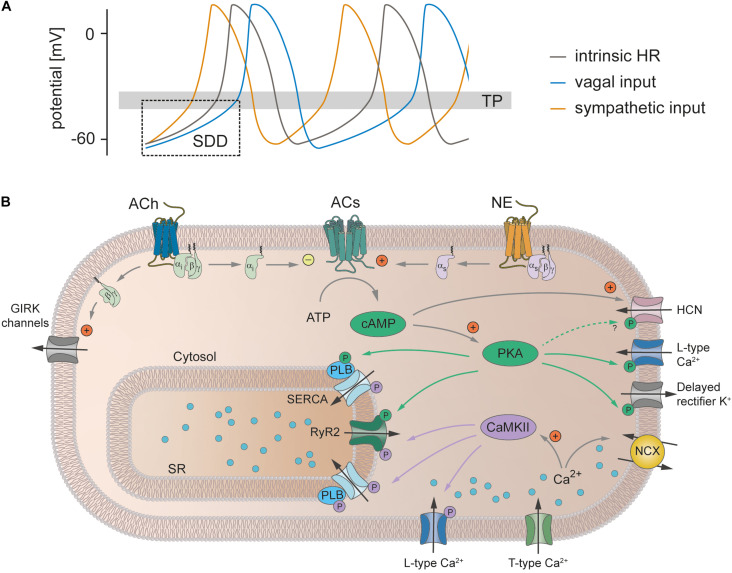
Heart rate regulation by the autonomic nervous system. **(A)** SAN cell action potentials. The intrinsic action potential rate is depicted in gray. Activity of the sympathetic nervous system (orange) leads to a steeper SDD, decreases the time to reach the threshold potential (TP) for the next action potential thereby increasing action potential frequency and consequently also heart rate (positive chronotropic effect). Vagal input flattens SDD, lowers the maximum diastolic potential and thereby increases the time to reach the TP. Action potential frequency and hence HR decrease (negative chronotropic effect). **(B)** Signaling cascades underlying the chronotropic effect. Release of norepinephrine (NE) from sympathetic nerve terminals leads to activation of Gs protein-coupled β-receptors. Subsequent stimulation of adenylyl cyclases (ACs) increases the cytoplasmic concentration of cAMP. cAMP directly facilitates the opening of HCN channels and activates PKA, which in turn phosphorylates various proteins (indicated by green arrows and green circles), thereby increasing their activity. In addition, sympathetic activity increases the intracellular Ca^2+^ concentration, which increases the activity of NCX and CaMKII. This in turn phosphorylates various target proteins (indicated by purple arrows and circles). Taken together, this steepens the SDD, increases the repolarization rate and thereby increases the action potential frequency (see text for further details). In contrast, the release of acetylcholine (ACh) during vagal activity leads to the activation of Gi protein-coupled M-receptors. ACs are inhibited by the Gi protein and thus the opposite effect of sympathetic activation unfolds. In addition, the β/γ-subunit activates GIRK channels that make the maximum diastolic potential more negative. Abbreviations: ACh, acetylcholine; ACs, adenylyl cyclases; ATP, adenosine triphosphate; CaMKII, Ca^2+^/calmodulin-dependent protein kinase II; GIRK, G protein-coupled inwardly rectifying potassium channels; HCN, hyperpolarization-activated cyclic nucleotide-gated cation channel; NE, norepinephrine; PKA, protein kinase A; PLB, phospholamban; RyR2, ryanodine receptor 2; SERCA, sarco-/endoplasmic reticulum Ca^2+^ ATPase; SR, sarcoplasmic reticulum.

The ANS consists of the sympathetic nervous system and parasympathetic nervous system. Both branches of the ANS innervate the SAN (Pauza et al., 2014) and activate intracellular signal transduction cascades in SAN pacemaker cells that regulate HR (Figure 3). Following activation of the sympathetic nervous system, the neurotransmitter norepinephrine is released from nerve terminals and activates G_*s*_ protein-coupled receptors. The subsequent stimulation of adenylyl cyclases increases the cytoplasmic concentration of the second messenger cAMP (Behar et al., 2016). cAMP binds to a variety of target proteins in the cell, which finally results in acceleration of SDD and consequently the firing rate of pacemaker cells and HR increase (positive chronotropic effect). In contrast, release of acetylcholine from parasympathetic nerve terminals inhibits adenylyl cyclase activity via activation of G_*i*_ protein-coupled receptors, followed by a reduction in the cytoplasmic cAMP concentration. In addition, acetylcholine-dependent G_β_.γ__ signaling activates G protein-coupled inwardly rectifying potassium channels (GIRK1 and GIRK4) that conduct an outward current (I_K,ACh_) which leads to membrane hyperpolarization. Together, this reduces the maximum diastolic potential and slope of SDD and as a consequence the firing rate and HR decrease (negative chronotropic effect). However, the exact signaling pathway by which alterations in intracellular cAMP mediate an increase or decrease in the firing frequency is still incompletely understood.

HCN4 channels, the main HCN channel isoform in the SAN, display several characteristics which make them an ideal target for HR regulation by the ANS. First, the channels are opened by hyperpolarization and conduct a depolarizing inward current (I_f_) throughout the time course of SDD, i.e., at membrane potentials negative to the channel’s reversal potential (Mangoni and Nargeot, 2008; Biel et al., 2009). Second, activity of HCN4 is directly regulated by binding of cAMP (DiFrancesco and Tortora, 1991) to a CNBD (Zagotta et al., 2003) in the intracellular C-terminus of the channel (cAMP-dependent regulation, CDR) and is, hence, tightly controlled by the ANS. Consequently, it has been postulated for a long time that CDR of HCN4 mediates the regulation of HR by the ANS. This hypothesis assumes that upon activation of the sympathetic nervous system, a cAMP-dependent increase in the I_f_ current is responsible for the acceleration of SDD that increases the firing rate of pacemaker cells and consequently the HR (Brown et al., 1979). On the other hand, a decrease in cAMP caused by activation of the parasympathetic nervous system would reduce HCN4 activity and slow down HR. However, until now this concept could not be validated *in vivo* and therefore remained controversially discussed. Moreover, it has been shown that, especially in the mouse SAN, the gating kinetics of HCN channels are much too slow to open and close the channels during successive action potentials, because the very high sinus rhythm and thus the firing rate of murine SAN pacemaker cells are markedly faster than the channel kinetics (Fenske et al., 2011, 2020; Hennis et al., 2021; Peters et al., 2021). As a result, HCN4-mediated current will manifest mainly as a nearly time-independent but bidirectionally flowing background current during the pacing cycle (Figure 2E), strongly arguing against a role for HCN4 in adjusting the slope of SDD and altering HR.

## Role of HCN4 in HR Regulation: Insights From Mouse Models and Human Patients

In the past 20 years, different groups have been investigating several mouse models targeting HCN4 (Table 1; Bucchi et al., 2012; Herrmann et al., 2012). The findings of these studies led to conflicting hypotheses about the role of HCN4 CDR in HR regulation. Stieber et al. (2003) created HCN4 knockout mice by generating a non-functional construct lacking the pore, which prevents translation of a functional protein and thereby results in complete absence of HCN4. Loss of HCN4 in these mice led to embryonic lethality due to a strongly diminished I_f_ during cardiac development. By studying embryonic HCN4^–/–^ hearts and isolated cardiomyocytes the authors found that HR was markedly reduced by about 40% and that HR and action potential firing rate could not be accelerated by cAMP. Surprisingly, a further study revealed that mice carrying only a point mutation in the CNBD of HCN4 also die during embryonic development (Harzheim et al., 2008). A single amino acid exchange (R669Q), which results in abolished CDR but otherwise unaltered channel function, caused embryonic lethality. This indicates that binding of cAMP to HCN4 is a general pre-requisite for the physiological function of the channel. Furthermore, the authors reported significantly reduced HRs and absent responses to catecholaminergic stimulation in embryonic hearts. Taken together, these studies suggest an important role of HCN4 CDR in mediating the chronotropic effect, although insights are restricted to findings from embryonic states in which the SAN and cardiac conduction system are not yet fully developed.

**TABLE 1:** Phenotypic manifestation of HCN4 mouse models.

References	Model	Tissue specificity	Baseline	Stimulation
**HCN4 knockout**				
Stieber et al. (2003)	HCN4 knockout	Global	Embryonic lethality	
		Cardiac specific	Embryonic lethality	
			Isolated embryonic cardiomyocytes: immature pacemaker potentials	cAMP: no increase in firing rate
			Isolated embryonic hearts: beating rate ↓	cAMP: no increase in beating rate
Herrmann et al. (2007)	HCN4 knockout	Global inducible, tamoxifen	Isolated SAN cells: large fraction of quiescent cells with hyperpolarized membrane potential	Iso: quiescent SAN cells rescued
			Isolated hearts: sinus pauses	
			*In vivo* adult mice: sinus pauses, HR unchanged	Iso: preserved max. HR, preserved chronotropic effect Carbachol or CCPA: HR ↓
Hoesl et al. (2008)	HCN4 knockout	Conduction system specific inducible, tamoxifen	Isolated SAN cells: large fraction of quiescent cells with hyperpolarized membrane potential	
			*In vivo* adult mice: sinus pauses, HR unchanged	Iso: preserved max. HR, preserved chronotropic effect Carbachol or CCPA: HR ↓
Baruscotti et al. (2011)	HCN4 knockout	Cardiac specific inducible, tamoxifen	Isolated SAN cells: firing rate ↓	Iso: max. firing rate ↓, preserved chronotropic action
			*In vivo* adult mice: bradycardia, AV-blocks, sinus arrest	Iso: max. HR ↓, preserved chronotropic effect
Mesirca et al. (2014)	hHCN4-AYA dominant-negative	Cardiac specific inducible, dox-withdrawal	Isolated SAN cells: firing rate ↓	Iso: max. firing rate ↓, preserved chronotropic action
			*In vivo* adult mice: bradycardia, sinus pauses, AV blocks, ventricular tachycardia	Iso: max. HR ↓, preserved chronotropic effect
**HCN4 overexpression and knockdown**				
Kozasa et al. (2018)	HCN4 overexpression	Global HZ without dox	Embryonic lethality (homozygous with and w/o dox)	
			Isolated SAN cells: firing rate unchanged	ACh: beating rate reduction ↓
			*In vivo* adult mice: HR unchanged, HRV ↓	Iso: preserved max. HR VNS: beating rate reduction ↓
	HCN4 knockdown	Global inducible, HZ with dox	Isolated SAN cells: firing rate ↓	ACh: beating rate reduction ↑
			*In vivo* adult mice: bradycardia, sinus arrhythmia, HRV ↑	Iso: preserved max. HR VNS: beating rate reduction ↑
**cAMP-insensitive HCN4 channels**				
Harzheim et al. (2008)	HCN4 R669Q knock-in	Global	Embryonic lethality (homozygous)	
			Isolated embryonic cardiomyocytes: firing rate ↓	
			Isolated embryonic hearts: beating rate ↓	Iso: absence of chronotropic effect
			Isolated adult hearts (HZ): unchanged beating rate	
			*In vivo* adult mice (HZ): unchanged HR	
Alig et al. (2009)	hHCN4-573X transgenic	Cardiac specific inducible, dox-withdrawal	Isolated SAN cells: quiescent and intermittent beating cells, beating rate ↓	Iso: rescues quiescent and interm beating cells, max. beating rate ↓, preserved chronotropic action
			*In vivo* adult mice: bradycardia, HR regulation preserved	
Fenske et al. (2020)	HCN4FEA Y527F, R669E, T670A knock-in	Global	Isolated SAN cells: cells alternate between firing and non-firing mode	Iso: time spent in non-firing mode ↓, preserved chronotropic action
			Isolated hearts: beating rate ↓	VNS: sinus pauses
			*In vivo* adult mice: bradycardia, sinus dysrhythmia, preserved chronotropic effect	

*Abbreviations: ACh, acetylcholine; cAMP, cyclic adenosine monophosphate; CCPA, 2-Chloro-N6-cyclopentyladenosine; dox, doxycycline; HR, heart rate; HZ, heterozygous; HRV, heart rate variability; Iso; isoproterenol; max., maximum; SAN, sinoatrial node; VNS, vagus nerve stimulation; ↓ decreased compared to WT condition, ↑ increased compared to WT condition.*

In order to overcome the limitations caused by embryonic lethality, Baruscotti et al. (2011) generated a mouse model in which cardiac-specific knockout of HCN4 was achieved in a temporally controlled manner, thereby enabling investigation of adult mice lacking HCN4 channels in the heart. In these mice, loss of HCN4 led to progressively developing bradycardia and AV block, which finally resulted in sinus arrest and cardiac death. However, HR response to beta-adrenergic stimulation was not affected by the tamoxifen-induced knockout of HCN4. In 2014, the findings were supported by a further study investigating heart-specific silencing of I_f_ by transgenic expression of a dominant-negative, non-conductive HCN4-channel subunit (hHCN4-AYA) in adult mice (Mesirca et al., 2014). The animals showed significant SAN dysfunction reflected by frequent sinus pauses and reduced HR, while sympathetic regulation of HR was unaltered. Unexpectedly, another study reported that diminished I_f_ due to a comparable approach of tamoxifen-induced, global knockout of HCN4 gave rise to cardiac arrhythmia in the form of recurrent sinus pauses, whereas the mice showed no signs of bradycardia under baseline conditions (Herrmann et al., 2007). However, HR acceleration induced by exercise or injection of isoproterenol was normal, which demonstrates preserved beta-adrenergic HR regulation also in these animals. Significantly lower HRs were only detected after application of carbachol or CCPA, an A1 adenosine receptor agonist, indicating overshooting parasympathetic responses of the SAN in the absence of HCN4. On the single cell level, knockout of HCN4 resulted in a quiescent phenotype of pacemaker cells characterized by a hyperpolarized maximum diastolic potential that was reversible upon application of isoproterenol. A further study by the same group, in which HCN4 was deleted explicitly in pacemaker cells of the sinoatrial and atrioventricular node, confirmed these results (Hoesl et al., 2008). Kozasa et al. (2018) investigated the contribution of HCN4 to the autonomic regulation of the SAN by transgenic overexpression or knockdown of HCN4 channels in mice. Surprisingly, overexpression of HCN4 did not induce tachycardia, but reduced HR variability, possibly due to excessively attenuated ANS input. This was evident in HR histograms derived from 24 h telemetric electrocardiogram (ECG) recordings. Histograms from HCN4-overexpressing mice were characterized by a unimodal distribution with a single peak in the intermediate frequency range and a symmetrically reduced total HR range. In contrast, HRs in WT histograms were bimodally distributed with a considerably broader frequency range. Furthermore, conditional knockdown of HCN4 induced pronounced bradycardia and gave rise to sinus arrhythmia and enhanced parasympathetic responses to cervical vagus nerve stimulation. Following injection of isoproterenol, bradycardia induced by cervical vagus nerve stimulation was attenuated in HCN4-overexpressing mice. Remarkably, neither overexpression nor knockdown of HCN4 altered the HR response of the SAN to beta-adrenergic stimulation. Taken together, this study suggests that HCN4 is responsible for stabilizing the spontaneous firing of the SAN mainly by attenuating the parasympathetic response. Thus, HCN4 channels protect the SAN network during parasympathetic regulation while the cAMP-dependent activation of HCN4 seems to enhance this protective effect. A few years ago, further insight into CDR of HCN4 was provided by Baruscotti et al. (2017), who identified the first gain-of-function mutation in HCN4 in a patient with inappropriate sinus tachycardia. The R524Q mutation is located in the C-linker, a region that couples cAMP binding to channel activation. Heterologous expression of R524Q mutant HCN4 channels revealed increased sensitivity to cAMP, and rat neonatal cardiomyocytes transfected with the mutant channel construct displayed enhanced spontaneous beating rates. Accordingly, the symptoms in human patients included prolonged periods of sinus tachycardia and frequent palpitations at rest and during exercise. This suggests that overactive CDR of HCN4 may lead to excessive responses of the SAN network to sympathetic stimulation, but unfortunately these observations were not further investigated mechanistically as no mouse model with this particular mutation is available to date.

To study the role of HCN4 CDR more specifically, a transgenic mouse model was generated by Alig et al. (2009). The mice were created with a mutation (573X) that was initially identified in a human patient with sinus node dysfunction (Schulze-Bahr et al., 2003). The structure of HCN4 is highly conserved between different species, including mice and humans, which in principle justifies the use of the mouse as a model organism for the study of HCN4 CDR. The mutation results in a truncation of the C-terminus of HCN4 that includes the CNBD and thereby causes cAMP insensitivity of the channel. Transgenic, cardiac-specific overexpression of the mutant construct (hHCN4-573X) in adult mice suppressed cAMP sensitivity of HCN4 in a dominant-negative manner. The cardiac phenotype of these mice was characterized by a marked reduction in HR at rest and during exercise, while the relative range of HR regulation was unchanged. On the single cell level, the mutation caused a heterogeneous phenotype with isolated pacemaker cells being arrhythmic, alternating between spontaneous firing and subthreshold membrane potential oscillations, or completely lacking electrical automaticity. These observations widely match with the symptoms of the human patient, which include bradycardia and chronotropic incompetence due to idiopathic sinus node dysfunction (Schulze-Bahr et al., 2003). The findings provide important insights into the physiological role of HCN4 in the SAN and further support the theory that HR regulation by the ANS is not mediated by CDR of HCN4. However, the mutation in this model causes a C-terminal truncation involving a total of 630 amino acids. Since the sequence of the CNBD comprises only 119 amino acids (Ludwig et al., 1999), it is obvious that the truncation eliminates not only the CNBD but also many other structural domains, including potential phosphorylation sites and binding domains for various modulators in addition to cAMP. Therefore, the cardiac phenotype of the mice cannot be directly attributed to absent CDR of HCN4.

To particularly investigate the physiological implication of HCN4 CDR, our group created a knock-in mouse model with two amino acid exchanges in the CNBD and one in the C-linker, which result in loss of CDR of the channel while embryonic lethality is prevented (HCN4FEA mouse line) (Fenske et al., 2020). These mice displayed pronounced bradycardia, whereas the entire range of HR regulation was preserved. The findings strongly support the theory that, in contrast to previous assumptions, CDR of HCN4 is not required for the classical chronotropic effect. In addition, several other human HCN4 channelopathies have been reported that result in loss of HCN4 function (Verkerk and Wilders, 2015). The mutations lead to diverse cardiac syndromes, the majority of which include bradycardia. However, in some of these channelopathies HR modulation by beta-adrenergic stimulation is preserved. Unfortunately, a straight forward interpretation of these cardiac phenotypes on HCN4 function is compromised by the fact that all patients identified so far display heterozygous mutations and thus also express an unmodified copy of HCN4.

In summary, evidence is growing that the role of HCN4 in the SAN is different to the originally postulated HR regulation by the ANS and that the classical chronotropic effect is mainly carried out by pacemaker mechanisms other than HCN4. Instead, it seems more likely that HCN4 is involved in setting the intrinsic HR (i.e., HR in the absence of autonomic regulation) and, in particular, in determining the lower part of the HR range. Furthermore, the channel appears to exert a protective effect on the SAN network especially during parasympathetic activity, thereby suppressing bradycardia. In contrast, HR modulation by the ANS is still possible in the absence of HCN4. It remains unclear why a minor part of the mouse studies did not report occurrence of bradycardia after deletion of HCN4 (Herrmann et al., 2007; Hoesl et al., 2008). Possible explanations might include that some HCN4 channels remain present after application of tamoxifen due to insufficient Cre activation. Furthermore, differences in the genetic background of the (sub-) strains of mice used in the studies could also contribute to these functional discrepancies (Bucchi et al., 2012).

## Non-Firing Pacemaker Cells in the Sinoatrial Node

Several mouse studies have shown that inhibition of various components involved in cardiac automaticity gives rise to the presence of quiescent states in isolated pacemaker cells. For example, pacemaker cells isolated from atrial-specific NCX knockout mice are completely quiescent, which leads to intermittent burst pacemaker activity of the SAN network. This is characterized by frequent, short pauses of a few seconds, reminiscent of human sinus node dysfunction and “tachy-brady” syndrome (Torrente et al., 2015). Furthermore, as outlined above, knockout of HCN4 as well as truncation of the HCN4 C-terminus cause quiescent phenotypes of isolated SAN pacemaker cells (Herrmann et al., 2007; Alig et al., 2009). However, it has only recently been discovered that non-firing pacemaker cells are also present and functionally relevant *in vivo* in the intact SAN of WT animals, especially during HR regulation by the ANS (Fenske et al., 2020). These findings reported by our group revealed that HCN4 channels indeed play an important role in HR regulation, but that this is based on a completely different mechanism than originally postulated. In this study, we found that isolated pacemaker cells expressing cAMP-insensitive HCN4FEA channels, but also WT cells, can spontaneously switch into a non-firing mode that is characterized by a hyperpolarized membrane potential and lasts for up to 1 min. Non-firing was more pronounced in HCN4FEA cells but could also reliably be induced in WT cells by application of carbachol or TAT-TRIP8b_nano_, a synthetic peptide that prevents CDR in HCN channels (Saponaro et al., 2018). Strikingly, *ex vivo* confocal calcium imaging of intact SAN preparations revealed that non-firing pacemaker cells are also present in the intact SAN and significantly modify SAN network activity. This is in line with findings from another recent study which reported presence of markedly heterogeneous calcium signals in adjacent cells within the SAN network (Bychkov et al., 2020), indicating that not all pacemaker cells in the SAN generate full-scale action potentials at a given time.

What is the exact functional relevance of non-firing pacemaker cells in the SAN? From our experimental findings we derived the hypothesis that a tonic electrical interaction via gap junctions takes place between cells in the non-firing mode and neighboring cells in the firing mode. During non-firing, pacemaker cells are significantly more hyperpolarized than firing cells for a period of up to 1 min. Through tonic interaction, these cells act as “brakes” in the network of the SAN and inhibit the activity of neighboring pacemaker cells in the firing mode. This interaction lowers the maximum diastolic potential and slows down the SDD of the firing cells, and thus slows down basal HR. In addition, tonic inhibition seems to increase during vagal activity and decrease during beta-adrenergic stimulation, probably because more cells switch to the non-firing mode or firing mode, respectively. This process is very important for setting the intrinsic HR and stabilizing SAN network activity, but not for changing HR *per se*. However, because the mechanism is dependent on cAMP and CDR of HCN4, it also contributes substantially to the regulation of SAN network activity by the ANS. According to this new hypothesis, CDR of HCN4 determines the number of pacemaker cells in the non-firing mode and is essential to ensure a safe and stable transition between different HRs during sympathetic and/or vagal activity, while it appears to be particularly important in fine-tuning the HR-lowering effect of the parasympathetic nervous system. During high vagal activity, HCN4 effectively counteracts hyperpolarizing changes in the membrane potential. This effect is due to the well-known properties of HCN channels to dampen inhibitory and excitatory stimuli, thereby stabilizing the membrane potential (Robinson and Siegelbaum, 2003; Nolan et al., 2004; Narayanan and Johnston, 2008; Biel et al., 2009; Wahl-Schott et al., 2014). Via CDR of HCN4, the extent of this antagonizing effect, i.e., the gain of negative feedback, can be adjusted according to the situation, which effectively stabilizes HR. Conversely, in the absence of CDR, responses of the SAN to ANS activity are exaggerated which results in extreme HR fluctuations, sinus dysrhythmia and instability of mean HR [for review see also: (Hennis et al., 2021)].

It is known that, in general, inhibitory elements increase the stability of electrically active networks. For example, inhibitory interneurons in the brain provide a mechanism to balance the activity of otherwise unstable neuronal networks (Markram et al., 2004; Sadeh and Clopath, 2021). Our work shows that inhibitory control of excitability is also essential in the SAN to ensure a stable function of the pacemaker process and to protect the SAN network during ANS activity. Taken together, CDR counteracts parasympathetic override, inappropriate HR decreases and the occurrence of bradycardia, which is completely different to the originally postulated HR regulation by the ANS and the classical chronotropic effect (Kozasa et al., 2018; Fenske et al., 2020).

## Alternative Concepts for Classical Chronotropic Response

It is becoming more and more accepted that the role of HCN4 in the SAN is to set the intrinsic HR and stabilize the SAN network during input from the ANS. Therefore, the question arises as to which molecular component(s) of SAN pacemaking actually mediate(s) the chronotropic effect. It is well known that beta-adrenergic signaling in the SAN activates the stimulating adenylyl cyclase–cAMP–protein kinase A (PKA) cascade (Figure 3B; Behar et al., 2016). Besides direct effects, e.g., on HCN4, cAMP binds to and activates PKA, which in turn phosphorylates and activates numerous target proteins in the cell, many of them involved in pacemaker activity (MacDonald et al., 2020). These targets include L-type Ca^2+^ channels (van der Heyden et al., 2005), phospholamban (Vinogradova et al., 2010), RyR (Shan et al., 2010), delayed rectifier potassium channels (Lei et al., 2000), Na^+^/K^+^-ATPase (Gao et al., 1994), and HCN channels (Liao et al., 2010). In addition, there is also evidence that sympathetic stimulation activates Ca^2+^/calmodulin-dependent protein kinase II (CaMKII) (Wu et al., 2009; Grimm and Brown, 2010; Wu and Anderson, 2014), which shares a number of downstream targets with PKA, i.e., L-type Ca^2+^ channels (Vinogradova et al., 2000), phospholamban and RyR2 (Li et al., 2016). Accordingly, voltage-gated Ca^2+^ channels are a component of the *membrane clock* different to HCN4 that could possibly mediate the chronotropic effect. While a potential isoproterenol-induced effect on I_Ca,T_ in the SAN is not completely resolved (Hagiwara et al., 1988; Li et al., 2012), I_Ca,L_ is enhanced following PKA-dependent (van der Heyden et al., 2005; Mangoni and Nargeot, 2008) and CaMKII-dependent phosphorylation (Dzhura et al., 2000; Vinogradova et al., 2000; Mangoni and Nargeot, 2008). In line with this, knockout of L-type Ca_V_1.3 channels in mice slowed the firing rate of isolated SAN pacemaker cells and reduced the slopes of both the early and late phase of SDD (Mangoni et al., 2003; Baudot et al., 2020). However, it has been indicated that I_Ca,L_ augmentation alone is not sufficient to achieve the normal increase in action potential firing rate upon beta-adrenergic stimulation (Vinogradova et al., 2002; Lakatta et al., 2010).

It is therefore possible that modulation of the *calcium clock* could play a key role in HR regulation by the ANS (Figure 3B). In this concept, activation of PKA and/or CaMKII would increase LCRs from the SR due to (A) accelerated Ca^2+^ reuptake into the SR following either direct CaMKII-dependent stimulation of SERCA (Narayanan and Xu, 1997) or disinhibition of SERCA by PKA/CaMKII-dependent phosphorylation of phospholamban (Vinogradova et al., 2010; Li et al., 2016) and/or (B) increased release of Ca^2+^ from the SR due to phosphorylation of RyR2 by PKA and/or CaMKII (Vinogradova et al., 2002; Bers, 2006; Shan et al., 2010). This would lead to enhanced LCRs that also occur earlier in the pacemaker cycle. Since LCRs activate NCX by elevating intracellular Ca^2+^, the consequences would include an increase in depolarizing I_NCX_ current with an onset earlier in SDD, thereby accelerating SDD and reducing the pacemaker cycle length, i.e., increasing the firing frequency (Bogdanov et al., 2006; Maltsev and Lakatta, 2009; Lakatta et al., 2010).

There are several aspects that argue in favor of this theory. First, Förster resonance energy transfer (FRET) experiments showed that PKA activity in isolated SAN pacemaker cells is tightly linked to action potential firing rate in response to either adrenergic or cholinergic stimulation (Behar et al., 2016). In addition, beta-adrenergic stimulation of PKA activity enhances LCRs, whereas inhibition of PKA abolishes LCRs and significantly interferes with cellular automaticity (Vinogradova et al., 2006). Furthermore, it has been shown that also CaMKII activity is essential for stimulating LCRs to cause a physiological HR increase (Wu et al., 2009; Swaminathan et al., 2011). This indicates that (possibly common) downstream targets of PKA and CaMKII, which are attributable to the *calcium clock*, could be responsible for mediating the chronotropic response.

Second, there is evidence that the rate of refilling the SR Ca^2+^ storage during diastole has a direct influence on the chronotropic state of the SAN. It has been demonstrated that direct pharmacological inhibition of SERCA prolongs the spontaneous cycle length of isolated SAN pacemaker cells, whereas changes in PKA/CaMKII-dependent phospholamban phosphorylation, which lead to successive disinhibition of SERCA, are paralleled by a reduction of the LCR period and pacemaker cycle length (Vinogradova et al., 2010; Li et al., 2016).

Third, it has been indicated that modulation of RyR2 function is indispensable for physiological HR adaption (Vinogradova et al., 2006; Eschenhagen, 2010; Shan et al., 2010). Accordingly, when RyRs in SAN pacemaker cells are blocked by ryanodine, or when phosphorylation of RyR2 by PKA is genetically inhibited, augmentation of I_NCX_, acceleration of SDD and increase in firing frequency following beta-adrenergic stimulation are diminished (Rigg et al., 2000; Lakatta et al., 2010; Shan et al., 2010).

Fourth, there is evidence that the Na^+^-Ca^2+^ exchanger (NCX) plays a fundamental role in SAN automaticity and contributes significantly to the positive chronotropic modulation of the SAN (Zhou and Lipsius, 1993; Bogdanov et al., 2001, 2006). NCX knockout mice and numerical I_NCX_ and Ca^2+^ dynamics model simulations revealed decreased or completely abolished responsiveness to isoproterenol stimulation (Bogdanov et al., 2006; Gao et al., 2013; Maltsev et al., 2013), suggesting that I_NCX_ is a crucial contributor to the *fight-or-flight* response in the SAN.

In conclusion, it has become clear that HCN4 is not required for mediating the classical chronotropic effect, but rather contributes to determining the intrinsic HR and protecting the stability of the SAN network during ANS activity. Instead, it is more likely that one or several components of the *calcium clock* are the key mediators of HR regulation by the ANS. However, since there are numerous redundant processes involved in this cascade, with some of them possibly representing backup mechanisms to ensure proper chronotropic responses in the case of dysfunction of other components, the major contributor to HR adaption in the SAN remains yet to be identified.

## Author Contributions

KH, SF, and CW-S wrote the manuscript. SF and RR composed the figures. RR, CP, and MB revised the manuscript. All authors carefully revised the literature and approved the final version of the manuscript.

## Conflict of Interest

The authors declare that the research was conducted in the absence of any commercial or financial relationships that could be construed as a potential conflict of interest.

## Abbreviations

ANS: autonomic nervous system
CaMKII: Ca^2+^/calmodulin-dependent protein kinase II
cAMP: cyclic adenosine monophosphate
CNBD: cyclic nucleotide-binding domain
ECG: electrocardiogram
FRET: Förster resonance energy transfer
GIRK: G protein-coupled inwardly rectifying potassium channel
HCN channel: hyperpolarization-activated cyclic-nucleotide gated cation channel
HR: heart rate
LCR: local calcium release
NCX: sodium-calcium exchanger
PKA: protein kinase A
RyR: ryanodine receptor
SAN: sinoatrial node
SDD: slow diastolic depolarization
SERCA: sarco-/endoplasmic reticulum Ca^2+^ ATPase
SR: sarcoplasmic reticulum
WT: wild type.
